# Multiscale Modeling of Red Blood Cell Mechanics and Blood Flow in Malaria

**DOI:** 10.1371/journal.pcbi.1002270

**Published:** 2011-12-01

**Authors:** Dmitry A. Fedosov, Huan Lei, Bruce Caswell, Subra Suresh, George E. Karniadakis

**Affiliations:** 1 Division of Applied Mathematics, Brown University, Providence, Rhode Island, United States of America; 2 Institute of Complex Systems and Institute for Advanced Simulation, Forschungszentrum Jülich, Jülich, Germany; 3 School of Engineering, Brown University, Providence, Rhode Island, United States of America; 4 Department of Materials Science and Engineering, Massachusetts Institute of Technology, Cambridge, Massachusetts, United States of America; Medical College of Wisconsin, United States of America

## Abstract

Red blood cells (RBCs) infected by a *Plasmodium* parasite in malaria may lose their membrane deformability with a relative membrane stiffening more than ten-fold in comparison with healthy RBCs leading to potential capillary occlusions. Moreover, infected RBCs are able to adhere to other healthy and parasitized cells and to the vascular endothelium resulting in a substantial disruption of normal blood circulation. In the present work, we simulate infected RBCs in malaria using a multiscale RBC model based on the dissipative particle dynamics method, coupling scales at the sub-cellular level with scales at the vessel size. Our objective is to conduct a full validation of the RBC model with a diverse set of experimental data, including temperature dependence, and to identify the limitations of this purely mechanistic model. The simulated elastic deformations of parasitized RBCs match those obtained in optical-tweezers experiments for different stages of intra-erythrocytic parasite development. The rheological properties of RBCs in malaria are compared with those obtained by optical magnetic twisting cytometry and by monitoring membrane fluctuations at room, physiological, and febrile temperatures. We also study the dynamics of infected RBCs in Poiseuille flow in comparison with healthy cells and present validated bulk viscosity predictions of malaria-infected blood for a wide range of parasitemia levels (percentage of infected RBCs with respect to the total number of cells in a unit volume).

## Author Summary

One of the most severe forms of cerebral malaria is caused by the parasite *Plasmodium falciparum*. During its development inside the host red blood cell it causes major mechanical and biochemical changes to the cell and can even alter its biconcave shape. The two main mechanical modifications are significant stiffening of the cell and increased cytoadherence to the arterial wall and other cells. These two effects can lead to dramatic flow modifications of infected blood, especially in the smallest vessels where occlusions are possible. In this work we combine a first-principles mathematical approach together with single-cell measurements to model seamlessly the infected and healthy red blood cells as well as the plasma flow, and predict their collective behavior in blood flow. Through systematic parallel simulations we quantify the mechanical and rheological properties of infected blood in malaria for a wide range of parasitemia levels, investigating in particular the effect of temperature.

## Introduction

Malaria is a disease caused by a *Plasmodium* parasite, which infects red blood cells (RBCs). In particular, *Plasmodium falciparum* (Pf) causes one of the most serious forms of malaria resulting in a nearly million deaths per year. Pf-parasitized red blood cells (Pf-RBCs) experience progressing changes in their mechanical and rheological properties as well as in their morphology [1], [2] during intra-erythrocytic parasite development, which includes three stages from the earliest to the latest: ring
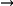
trophozoite
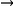
schizont.

A healthy human RBC is a soft biconcave capsule with an average diameter of 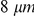. The RBC membrane consists of a lipid bilayer with an attached spectrin network known as the cytoskeleton. Progression through the stages of the parasite development in malaria leads to considerable stiffening of Pf-RBCs as found in optical tweezers stretching experiments [3] and in diffraction phase microscopy by monitoring the membrane fluctuations [4]. Pf development also results in vacuoles formed inside of RBCs reducing the cell volume. Thus, Pf-RBCs at the final stage (schizont) often show a “near spherical” shape, while in the preceding stages maintain their biconcavity. These changes greatly affect the rheological properties and the dynamics of Pf-RBCs, and may lead to obstruction of small capillaries [2] impairing the ability of RBCs to circulate. An additional significant stiffening of Pf-RBCs may occur during fever periods of the disease as shown in recent experiments [5], where the temperature was increased from the physiological value (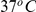) to the febrile (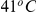).

Freely circulating Pf-RBCs are virtually invisible to the immune system, but are likely to be destroyed in the spleen [6]. As a survival mechanism for the successful intra-cell development, Pf parasites expose adhesive proteins on the RBC membrane surface to facilitate adhesion to the vascular endothelium and also to other healthy or Pf-RBCs. This mechanism allows for further progression of malaria, but it may severely disrupt normal blood flow and result in blockages of small vessels in specific organs, e.g. in cerebral malaria [7].

Numerical simulations may be used for qualitative as well as quantitative predictions of blood flow properties and behavior in malaria. Recent progress in numerical modeling of soft matter, and RBCs in particular, allows us to model blood flow in microvessels at sufficient detail. Examples include RBC modeling at the continuum level [8], [9] and at the mesoscopic level [10]–[13]. Different models may focus on different properties of RBCs; however, to adequately capture mechanical and rheological RBC properties, numerical models have to represent accurately the membrane elastic and viscous properties, bending resistance, and the viscosity contrast between the external and internal fluids; see [13] for details.

The first results of multiscale modeling Pf-RBCs were reported in [14] addressing blood flow resistance and adhesive dynamics of individual Pf-RBCs. Here we extend this study and focus on comparisons with different experimental data and, in particular, we investigate the effect of temperature. Our objective is to conduct the most complete validation of the RBC model to date against a diverse set of experimental results both on the rheology and dynamics, and to identify the possible limitations of this purely mechanistic model for future improvements. To this end, we present here extensive results on the RBC membrane fluctuations as well as validated predictions of the bulk viscosity with parasitemia levels up to 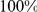. The modeled RBCs are represented by a network of viscoelastic springs in combination with bending energy and constraints for surface-area and volume conservation. The model is multiscale since the RBC can be represented on the spectrin level, where each spring in the network corresponds to a single spectrin tetramer with the length of approximately 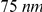. On the other hand, the RBC network can also be highly coarse-grained up to the spring lengths of about 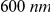. These sub-cellular scales are coupled to the vessel size scales of about ten microns. The membrane macroscopic properties are uniquely related to its microscopic parameters without adjustment of the model parameters. The multiscale RBC model was shown to accurately reproduce realistic mechanical and rheological properties and dynamics of healthy RBCs [13].

The paper is organized as follows: In the next section we give an overview of the RBC model including inter-cell interactions and scaling to physical units. In section 3 we first present simulations of the mechanical response, followed by rheological results, and blood flow results. We conclude in section 4 with a discussion focusing on the possible limitations of the present study.

## Methods

RBCs and their enclosing and surrounding fluids are all modeled within the Dissipative Particle Dynamics (DPD) method framework [15]. DPD is a mesoscopic particle-based simulation technique, where each particle represents a *cluster* of atoms or molecules rather than an individual atom. DPD particles interact through pairwise soft forces and move according to the Newton's second law of motion. A detailed description of the DPD method can be found elsewhere [15], [16]. Further, we provide a short description of the RBC model [13], [17] and of the inter-cell adhesion interactions.

### RBC membrane

The RBC membrane is represented by 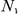 DPD particles with coordinates 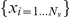 which are vertices of a two-dimensional triangulated network on the RBC surface [10], [13], [17]. The network has a fixed connectivity with the energy as follows
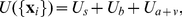
(1)where 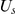 is the spring's potential energy, 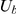 is the bending energy, and 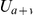 corresponds to the area and volume conservation constraints. The 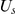 contribution provides membrane elasticity similar to that of a spectrin network of RBC membrane. A “dashpot” is attached to each spring, and therefore, the spring forces are a combination of conservative elastic forces and dissipative forces, which provide network viscous response similar to RBC membrane viscosity. The bending energy mimics bending resistance of the RBC membrane, while the area and volume conservation constraints mimic area-incompressibility of the lipid bilayer and incompressibility of a cytosol, respectively. Below, these energies are described in detail.

The network nodes are connected by 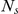 springs with the potential energy as follows
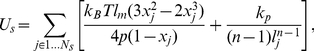
(2)where 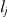 is the length of the spring 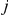, 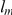 is the maximum spring extension, 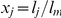, 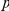 is the persistence length, 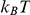 is the energy unit, 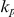 is the spring constant, and 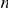 is a power. The above equation includes the attractive wormlike chain potential and a repulsive potential for 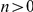 such that a non-zero equilibrium spring length can be imposed. The performance of different spring models for the RBC membrane was studied in [17] in detail.

To incorporate the membrane viscosity into the RBC model a dissipative force is introduced for each spring. Following the general framework of the fluid particle model [18] we can define dissipative 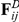 and random 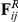 forces for each spring, where 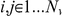 are a pair of two network vertices connected by a spring. Such forces satisfy the fluctuation-dissipation balance providing consistent temperature of the RBC membrane in equilibrium and are given by
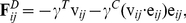
(3)

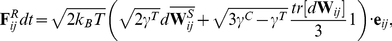
(4)where 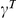 and 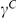 are dissipative parameters and the superscripts 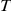 and 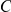 denote the “translational” and “central” components, 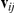 is the relative velocity of spring ends, 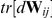 is the trace of a random matrix of independent Wiener increments 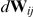, and 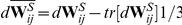 is the traceless symmetric part. Note that the condition 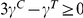 has to be satisfied.

The bending energy of the RBC membrane is given as follows
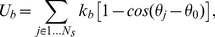
(5)where 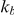 is the bending constant, 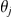 is the instantaneous angle between two adjacent triangles having the common edge 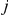, and 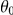 is the spontaneous angle.

In addition, the RBC model includes the area and volume conservation constraints with the corresponding energy given by
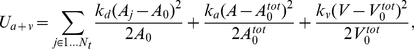
(6)where 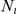 is the number of triangles in the membrane network, 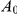 is the triangle area, and 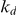, 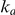 and 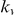 are the local area, global area and volume constraint coefficients, respectively. The terms 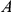 and 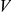 are the total RBC area and volume, while 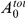 and 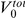 are the specified total area and volume, respectively. More details on the RBC model can be found in [13], [17], including aspects of coarse-graining of RBCs by varying 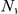 from the spectrin level (about 30,000 points) to the coarsest level (100 points).

### Membrane macroscopic properties

Extension of the linear analysis of [19] for a regular hexagonal network allows us to uniquely relate the model parameters and the network macroscopic elastic properties (shear, area-compression, and Young's moduli), see [13], [17] for details. The derived shear modulus of the membrane is given by
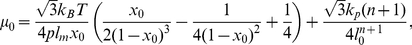
(7)where 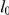 is the equilibrium spring length and 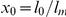. The area-compression 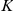 and Young's 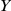 moduli are equal to 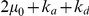 and 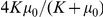, respectively.

The relation between the model bending coefficient 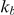 and the macroscopic bending rigidity 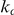 of the Helfrich model [20] can be derived as 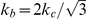 for a spherical membrane [17]. This expression describes bending contribution of the energy in equation (5), but may not fully represent actual bending resistance of the RBC membrane since membrane bending may also result in local in-plane deformations. The membrane shear viscosity 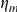 is related to the dissipative parameters 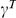, 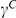 as 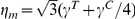. Since 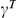 accounts for a larger portion of the viscous contribution, we set 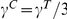 in all simulations, which satisfies the condition 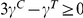 in equation (4).

In practice, the given macroscopic RBC properties 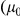, 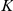, 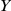, 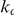, and 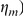 serve as an input to be used to calculate the necessary mesoscopic model parameters from the equations above without any manual adjustment. Thus, the spring parameters (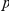 and 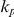) can be uniquely calculated for given 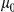 using equation (7) and the fact that the spring force 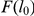 with the potential defined in equation (2) is equal to zero. Here, we assume that 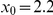 and 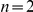 are constants (see [17]) because they contribute only within a non-linear deformation regime. The relations for the membrane bending rigidity and viscosity are rather straightforward, while the area and volume constraint coefficients can be set large enough to approximate incompressibility conditions for the membrane and inner cytosol.

A simulation of a RBC in equilibrium shows that the membrane may develop local bumps due to stress anomalies in a membrane triangulation since the membrane network consists of triangles whose edges have different lengths. Such local stress artifacts depend on the network regularity and the ratio of the membrane elastic and bending contributions given by the Föppl-von Kármán number 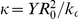, where 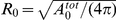. To eliminate the stress artifacts we employ a *“stress-free”* model [17] obtained by computational annealing, which assumes that the equilibrium length 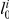 of each spring is equal to the edge length after triangulation for 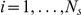. This also results in an individual maximum spring extension 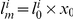. Then, the individual spring parameters (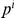 and 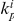) can be uniquely calculated for each spring and given 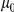 using equation (7) and the fact that the spring force 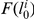 vanishes at 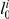. This modification provides a network free of local stress anomalies.

### RBC-fluid boundary conditions

Both internal and external fluids are simulated by a collection of free DPD particles and are separated by the RBC membrane through bounce-back reflections of them at a membrane surface. Moreover, a dissipative force between fluid particles and membrane vertices is set properly to account for the no-slip boundary conditions at the membrane surface. More details on boundary conditions can be found in [13], [17].

### Inter-cell adhesion interactions

The attractive cell-cell interactions are crucial to represent adhesion among cells in malaria. These forces are approximated phenomenologically with the Morse potential given by
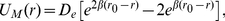
(8)where 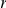 is the separation distance, 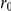 is the zero force distance, 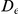 is the well depth of the potential, and 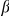 characterizes the interaction range. The Morse potential interactions are implemented between every two vertices of separate RBCs if they are within a defined potential cutoff radius 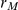. The Morse interactions consist of a short-range repulsive force when 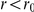 and of a long-range attractive force for 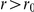. However, such repulsive interactions cannot prevent two RBCs from an overlap. To guarantee no overlap among RBCs we employ specular reflections of RBC vertices on membranes of other RBCs.

### Model and physical units scaling

The dimensionless constants and variables in the DPD model must be scaled with physical units. We define the length scale based on the cell diameter 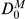 with 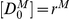, where the superscript 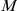 denotes “model” units and 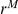 is the model length scale. A real RBC has an average diameter 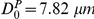 (superscript 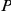 denotes “physical” or SI units), and therefore the following length scale is adapted
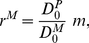
(9)where 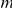 stands for meters.

A parameter which provides a scaling base and we are free to select is the imposed shear modulus 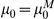 with 
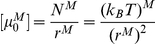
 (“
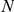
” denotes Newton) or equivalently the imposed Young's modulus 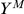. Therefore, we define the energy per unit mass (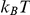) and the force unit scales as follows
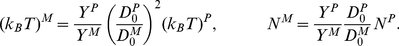
(10)After we determined the model energy unit (as an example for room temperature of 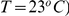, we can calculate the bending rigidity in model energy units corresponding to 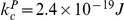.

The time scale is defined as
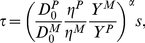
(11)where 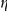 is the characteristic viscosity (e.g., solvent or membrane viscosity) and 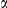 is a chosen scaling exponent similar to the power-law exponent in rheology.

The simulations with healthy RBCs employed the model with the following parameters: 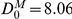, 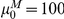, 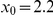, 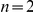, 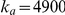, 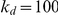, and 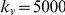. The energy unit 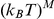 is equal to 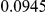 at 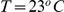 calculated according to the energy scale in equation (10). The shear moduli in the model units for other stages of the parasite development and temperatures we obtained by scaling 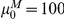 for healthy RBCs to the values calculated according to their relative ratio of the shear modulus with respect to that of a healthy RBC (see Tables 1 and 2).

**Table 1:** Shear moduli of healthy RBCs and Pf-RBCs in 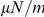 at 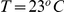.

Healthy	Ring	Trophozoite	Schizont
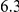	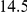	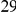	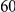 & 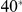

The “*” denotes a “near-spherical” RBC at the schizont stage.

**Table 2:** Shear moduli of healthy RBCs and Pf-RBCs in 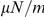 at the physiological and febrile temperatures obtained in [4].

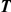	Healthy	Ring	Trophozoite	Schizont
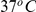	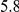	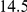	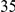	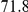
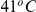	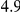	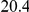	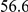	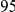

## Results

The mechanics of modeled Pf-RBCs is tested for various stages of parasite development and compared with the optical tweezers experiments of [3]. Rheological properties of Pf-RBCs at different stages and temperatures are probed by simulated twisting cytometry (STC), a numerical analog of the optical magnetic twisting cytometry (OMTC) [5], and by monitoring of Pf-RBC membrane fluctuations. The Pf-RBC dynamics in Poiseuille flow is simulated in a tube of diameter 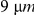. Finally, bulk-blood viscosity in malaria is predicted in shear flow simulations and is validated against experimental results [21] for parasitemia levels (percentage of infected RBCs with respect to the total number of cells in a unit volume) up to 100%.

### Increased stiffness Pf-RBCs

**Figure 1:**
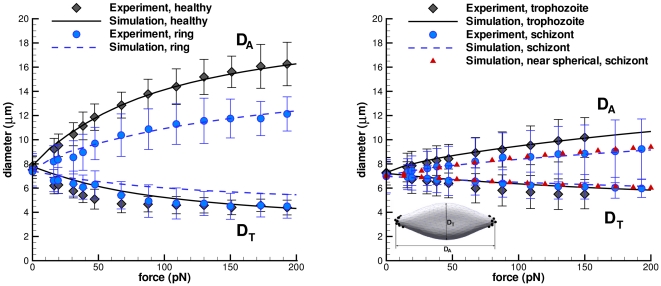
Stretching response of healthy RBCs and Pf-RBCs for different stages compared with the experiments in [**3**] at 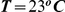 **.** 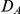
 and 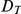 are the axial and transverse RBC diameters, respectively, as shown in the inset plot. The shear moduli of Pf-RBCs for different stages are given in Table 1. The bending stiffness and the membrane viscosity in all cases are set to 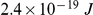 and 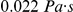, respectively.

Optical tweezers experiments on the Pf-RBC stretching [3] suggest gradual cell membrane stiffening during the consecutive stages of intra-erythrocytic parasite development. To mimic the experiments a modeled Pf-RBC (using 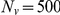 per Pf-RBC) undergoes stretching by applying a stretching force to 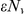 vertices on both ends of the cell along the negative and the positive directions, which is similar to the setup in [13], [14], [17]. The membrane area of the applied force is characterized by the fraction 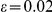 and is in agreement with the contact area of the attached silica bead with diameter 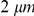 used in experiments. We compute the axial diameter 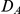 of a stretched RBC as 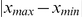, where 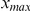 is the maximum position of all RBC vertices along the stretching direction 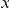, while 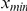 is the corresponding minimum position. The transverse diameter 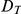 is calculated as 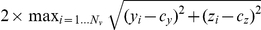, where 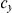, 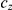 are the 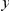 and 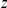 center of mass coordinates. Figure 1 shows a comparison of simulation results of healthy and Pf-RBCs at different stages with experiments; good agreement is obtained for the shear modulus values given in Table 1. The bending stiffness in all cases was set to that of healthy RBCs, 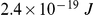, since its dependence for different stages of malaria is not known. The curve for the schizont stage marked “near-spherical” corresponds to stretching a membrane of ellipsoidal shape with the axes 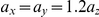. To match the experimental stress-strain response the membrane shear modulus of 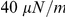 is chosen, which is smaller than that for the biconcave-shape simulation. The RBC model is able to accurately describe both linear and non-linear deformations of Pf-RBCs at different stages. In addition, these results emphasize that proper cell geometry representation is crucial in such tests, which is discussed in section.

### Pf-RBC rheology from magnetic twisting cytometry

Rheological measurements of Pf-RBC membrane properties provide a detailed description of the complex time-dependent membrane response. They are used to characterize viscoelastic membrane behavior associated with an interplay of membrane elastic and viscous properties. Experimental investigations of Pf-RBC rheology [5] employed OMTC, which is a technique where a response of a ferrimagnetic microbead (attached to the RBC top) to an oscillating magnetic field is measured. The corresponding numerical analog, STC, was first employed in [13] to probe membrane rheology of a healthy RBC by modeling an attached microbead subjected to an oscillating torque. The RBC-wall adhesion is simulated by keeping stationary 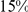 of vertices on the RBC bottom, while the RBC-bead adhesion is modeled by including several RBC vertices next to the microbead bottom into its rigid motion. A typical bead response to an oscillating torque shows the periodic displacement of the same frequency 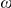 as the applied torque, but with a phase shift angle 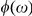 with respect to the latter. The angle 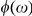 allows us to calculate components of the complex modulus as follows
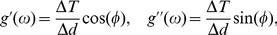
(12)where 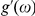 and 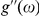 are the two-dimensional storage and loss moduli (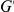 and 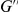 in 3D), and 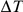 and 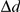 are the torque and bead displacement amplitudes, respectively.

**Figure 2:**
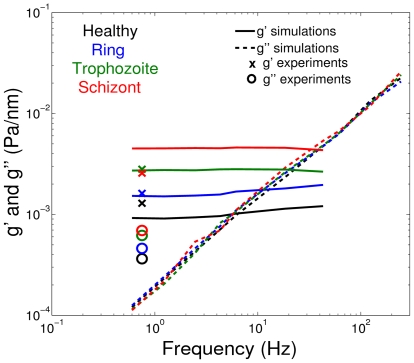
Components (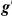 and 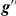) of the complex modulus for healthy RBCs and Pf-RBCs at different stages at 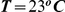 obtained from STC simulations. Experimental data [5] for the frequency 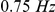 are drawn in symbols. The shear moduli of Pf-RBCs for different stages are given in Table 1. The bending stiffness and the membrane viscosity in all cases are set to 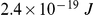 and 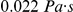, respectively.

Simulation results corresponding to STC for healthy RBCs in [13] revealed that the storage modulus (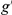) depends on the membrane elastic properties and bending rigidity, while the loss modulus (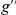) is governed by the membrane viscosity. The shear moduli of Pf-RBCs at 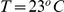 are set to the values given in Table 1, while the bending stiffness and the membrane viscosity are assumed to be 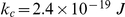 and 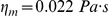, respectively, in all simulations. Figure 2 shows components of the complex modulus for healthy RBCs and Pf-RBCs at different stages. It also includes several data points for the frequency 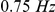 obtained in OMTC experiments [5]. The simulation results show the expected trend, i.e., an increase in 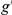 for the consecutive intra-erythrocytic stages since the shear modulus of the membrane is increased, while 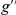 does not change for different stages because the membrane viscosity is kept constant. Agreement between STC and experiments is qualitative at best. Experimental data show an increase of 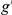 for the progressing stages of the parasite development, but it appears to be less pronounced than that in the STC simulations. Moreover, experiments show an increase in 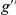 indicating a rise in the membrane or the internal fluid viscosity.

OMTC experiments [5] showed a strong dependence of membrane rheological properties on temperature for healthy RBCs and Pf-RBCs. Specifically, the increase of temperature from the physiological value 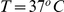 to the febrile 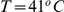 results in considerable stiffening of Pf-RBCs at later stages. Analogous conclusions were made in the experiments that observed membrane fluctuations [4]. Table 2 shows the shear moduli of healthy RBCs and Pf-RBCs for different temperatures obtained in the experiments of [4]. The dependence of other membrane properties (e.g., bending rigidity, membrane viscosity) on temperature is not known, and therefore we assume 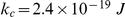 and 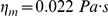 in the simulations that follow.

**Figure 3:**
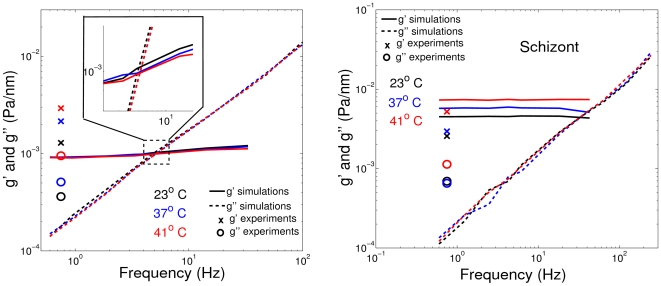
Components (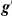 and 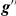) of the complex modulus for healthy RBCs (left) and Pf-RBCs at the schizont stage (right) for different temperatures. Experimental data [5] for the frequency 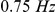 are drawn in symbols. The shear moduli of Pf-RBCs for different stages and temperatures are given in Tables 1 and 2. The bending stiffness and the membrane viscosity in all cases are set to 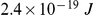 and 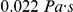, respectively.

Figure 3 presents components of the complex modulus for healthy RBCs (left) and Pf-RBCs at the schizont stage (right) for different temperatures. The change in the complex modulus for healthy RBCs at different temperatures appears to be relatively small in simulations. A small decrease in 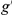 with temperature increase is found since the membrane shear modulus becomes slightly smaller as shown in Table 2. In contrast, the OMTC experiments show a gradual increase of 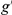 as temperature rises, which indicates progressive membrane stiffening. Another characteristic feature of the experimental data of [5] in Figure 3 (left) is the increase of the loss modulus 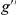 with increasing temperature indicating a rise in the membrane viscosity. The viscosity of liquids is known to decrease with temperature [22] suggesting an analogous behavior for the liquid-like lipid membrane of RBCs. Figure 3 (right) shows a gradual increase of the storage modulus with temperature increase in STC simulations. This is an expected trend since the membrane shear modulus is increased according to the data in Table 2. The correspondence between the experiments and the simulations is not satisfactory. The factors that may influence rheological membrane measurements are discussed in section suggesting that new experimental and modeling procedures are required in order to understand the complex membrane rheology in malaria and to reconcile the discrepancies found.

### Membrane fluctuations

Membrane fluctuations or “flickering” can be directly interpreted in terms of the cell membrane properties. Flickering of healthy and Pf-RBCs was measured in experiments using microrheology [23] by dynamical tracking of microbeads attached to the RBC surface and by diffraction phase microscopy [4], [24], where instantaneous RBC-height maps were obtained. These undulations can be used to derive the dynamic complex modulus similar to that described in the previous section.

#### Fluctuation maps and Pf-RBC membrane properties

**Figure 4:**
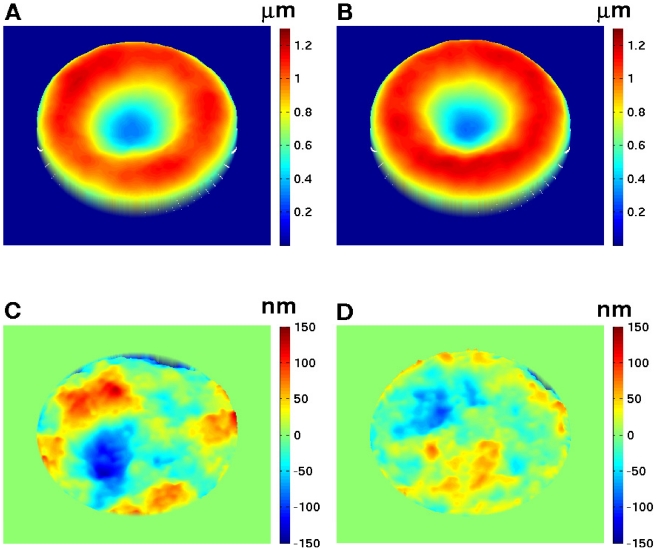
Instantaneous height and fluctuations of healthy RBC (A and C) and Pf-RBC in trophozoite stage (B and D) at 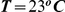 **.** A RBC is attached to a solid surface by fixing 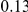 fraction of vertices on the RBC bottom. The instant fluctuation map is obtained by subtracting time-averaged cell shape from the instantaneous height map. Zero value in A and B corresponds to the half height of RBC. The shear moduli of Pf-RBCs for different stages are given in Table 1. The bending stiffness and the membrane viscosity in all cases are set to 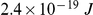 and 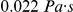, respectively.

Diffraction phase microscopy experiments [4] monitored the fluctuations of a RBC adhered to a solid surface by measuring instantaneous heights at different points of the cell surface. Adhesion of the modeled RBC to a solid surface is performed by fixing a fraction of vertices on the RBC bottom, while the other vertices are free to move. The cell is filled and surrounded by viscous fluids and the fluctuations on the RBC top are monitored in time. Figure 4 shows the instantaneous thickness and fluctuation maps of a healthy RBC (A and C) and a Pf-RBC in trophozoite stage (B and D). Both RBCs have the same equilibrium shape (A and B), while the shear elastic modulus was set to 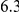 and 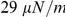 for the healthy and Pf-RBCs in trophozoite stage, respectively. The images C and D clearly indicate that the Pf-RBC has a smaller fluctuation amplitude than that of the healthy one in agreement with the results in [4].

**Figure 5:**
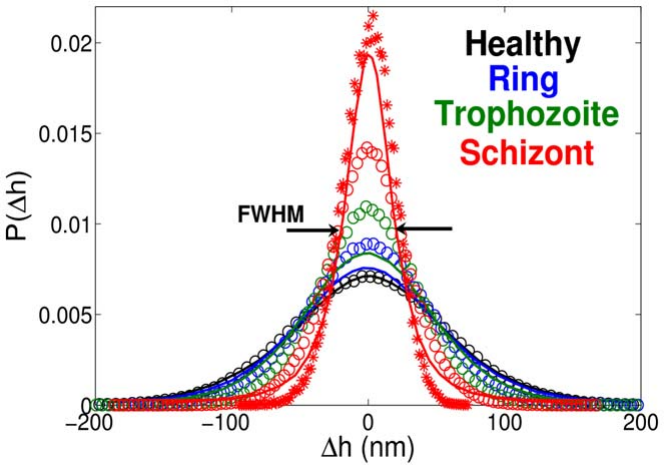
Membrane fluctuation distributions at different stages of Pf-RBCs monitored at room temperature 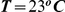 **.** The experimental data [4] are drawn with solid lines, simulations employing the biconcave RBC shape are plotted with circles, and a simulation with a nearly spherical shape is shown by “*”. FWHM identifies the full-width half-maximum value of the distribution curves. The shear moduli of Pf-RBCs for different stages are given in Table 1. The bending stiffness and the membrane viscosity in all cases are set to 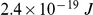 and 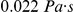, respectively.

A number of simulations was performed for different stages of the parasite development to identify dependence of the fluctuations on the membrane properties. Figure 5 presents membrane fluctuation distributions for different stages of Pf-RBCs at room temperature in comparison with the experiments [4]. The circles in Figure 5 correspond to the results of simulations employing a biconcave RBC shape with bending rigidity 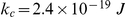. The strength of RBC adhesion here is characterized by the 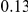 fraction of vertices on the RBC bottom held stationary, while the corresponding shear moduli of Pf-RBCs at room temperature are given in Table 1. Agreement between the distributions in experiments (solid lines) and simulations is found to be excellent for the case of healthy RBCs, while simulations for ring and trophozoite stages predict more narrow distributions than those in the experiments, and a wider distribution for the schizont stage. The curve in Figure 5 plotted with “*” symbols corresponds to a simulation employing a nearly spherical membrane (often observed in experiments) for the schizont stage, and yields a better agreement with the experiments. Hence, the effective geometry and local curvature may affect the fluctuation measurements. However, the observed discrepancies between the experiments and the simulations suggest that the shear modulus alone cannot provide an appropriate description of the fluctuations.

**Figure 6:**
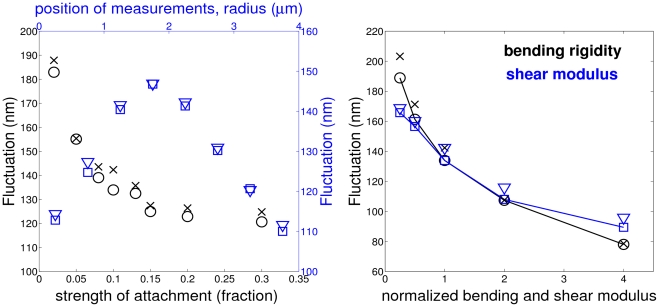
Sensitivity of membrane fluctuations. Sensitivity to the strength of attachment (vertex fraction) and the position of measurements (stripes of thickness 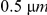 with different radii) (left). Dependence of fluctuations on the bending rigidity and the shear modulus normalized by healthy RBC values 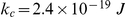 and 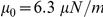 (right). 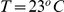. Black circles and blue squares correspond to the full-width half-maximum (FWHM) values measured directly from the simulated distributions, while black crosses and blue triangles are the FWHM values obtained from the fitted Gaussian distributions to the calculated distributions. The membrane viscosity in all cases is assumed to be 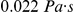.

Figure 6 shows dependence of fluctuation measurements on the simulated conditions (left) and the membrane properties (right). The black circles and blue squares in Figure 6 are the full-width half-maximum (FWHM) values obtained directly from the computed distributions, while the black crosses and blue triangles correspond to the FWHM values of the fitted Gaussian distributions to the simulated distributions, that are equal to 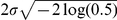, where 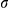 is the standard deviation. Figure 6 (left) shows that as the strength of RBC attachment characterized by the fraction of vertices held stationary decreases, the fluctuation distribution widens since the FWHM values increase. The adhesion strength may be difficult to control in experiments; however, our simulation results indicate independence of the fluctuation measurements on the adhesion strength if the fraction of the fixed vertices is greater than 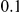. Furthermore, the simulation results in Figure 6 (left) show that the fluctuations may not be isotropic on the cell surface. The blue symbols are the FWHM measurements obtained for the circular areas of thickness 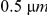 and different radii on the RBC top. Fluctuations appear to be smaller in the RBC center and on the side compared with a maximum in-between.

Figure 6 (right) presents dependence of the FWHM values on the membrane bending rigidity and the shear modulus normalized by their healthy RBC values 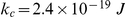 and 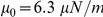. Here and further, the FWHM values in simulations are averaged over a RBC surface area of radius 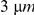 from the cell center. Both membrane properties strongly affect the values of fluctuations with a common trend: the stiffer the RBC, the smaller the fluctuation amplitudes characterized by FWHM values.

**Figure 7:**
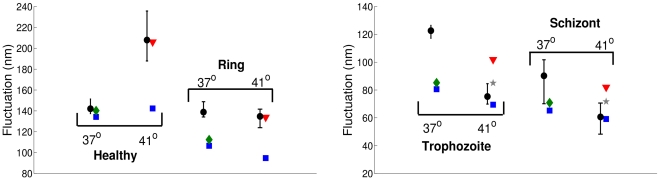
Pf-RBC full-width half-maximum (FWHM) values of fluctuation distributions for different stages of the parasite development at the physiological 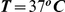 **and the febrile** 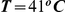 **temperatures.** Black circles are the median values of FWHM in experiments [4], while error bars correspond to standard deviation. Other symbols are the FWHM values in simulations. Blue squares assume the bending rigidity 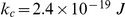, green diamonds 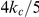, red triangles 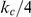, and gray stars 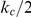. (The statistical error in all simulations is negligible.) The corresponding shear moduli are outlined in Table 2. The membrane viscosity in all cases is assumed to be 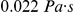.

Pf-RBCs become stiffer as the parasite develops, and they show a significant stiffening when the temperature is increased from the physiological value 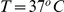 to the febrile 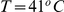 as observed in the experiments [4], [5]. In addition, the febrile temperature can lead to irreversible changes in the membrane properties in the later stages of Pf-RBCs, while healthy RBCs and those at the ring stage recover their elastic properties as temperature is lowered. Figure 7 shows the FWHM of thermal fluctuation distributions for different Pf-RBC stages at physiological and febrile temperatures. The shear moduli for different stages and temperatures are shown in Table 2. The FWHM value of healthy RBCs at physiological temperature is slightly lower in simulations (blue squares) than that in experiments (black circles), while at room temperature the agreement of fluctuation distributions is excellent (see Figure 5). Hence, the increase in temperature from 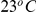 to 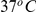 and the small decrease in the shear modulus from 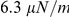 to 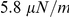 cannot fully explain the increase of fluctuations as the temperature is elevated from 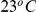 to 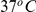. This suggests that there may be additional changes in the membrane properties (e.g., bending rigidity) or biochemical activities (e.g., metabolic) that influence the membrane undulations when the temperature is increased. An effective decrease in the membrane bending rigidity by about 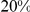 as temperature reaches 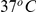 (shown by the green diamonds) provides good agreement between the simulations and the experiments. This seems to provide a plausible explanation since several experimental results on lipid vesicles [25], [26] show a slight decrease in bending rigidity with increasing temperature. However, as we increase temperature to the febrile value, the discrepancy between simulations (blue squares) and experiments (black circles) for healthy RBCs becomes more pronounced. This difference can be reconciled by an effective bending rigidity to be four times lower (red triangles) than 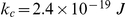 at room temperature, however such a sudden decrease in bending rigidity is not likely to occur.

The FWHM values of the Pf-RBCs in Figure 7 at physiological temperature are lower in simulations than those in experiments. This also indicates a complex dependence of membrane fluctuations on the membrane properties and potential metabolic activities. As an example, healthy and ring-stage RBCs at 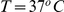 show comparable FWHM values in the experiments, while the corresponding shear modulus for the ring-stage is about 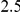 times higher than that of healthy RBCs. A decrease in the membrane bending rigidity is likely to take place for Pf-RBCs since the malaria parasite exposes intramembrane proteins that are known to affect membrane properties; however, it offers only partial explanation for the discrepancies found. Simulation results at the febrile temperature seem to show a better agreement with experiments for the later stages of parasite development. However, we did not find any consistent trend that would properly correlate the experimental FWHM values with membrane properties at different stages and temperatures.

These discrepancies suggest that the purely mechanistic model and interpretation may not provide a complete explanation for the experimental observations. Biological entities like Pf-RBCs may have ongoing active processes, which were not taken into account in either experiments or simulations. For instance, more recent experiments [27] with healthy RBCs clearly showed the presence of so-called *active fluctuations* due to metabolic activity, which are comparable to the passive thermal fluctuations. This is an indication that better-controlled experiments and more complex models that include such metabolic activity may be needed.

#### Complex modulus of Pf-RBCs

Membrane fluctuation measurements can be also used to infer the membrane's viscoelastic properties in terms of the dynamic complex modulus similar to that in the STC tests. In microrheology experiments [23] with healthy RBCs, microbeads attached to the membrane surface were tracked and the observed motion was used to compute the three-dimensional complex modulus 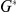 with components 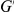 and 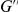, analogously to the two-dimensional version described in section.

**Figure 8:**
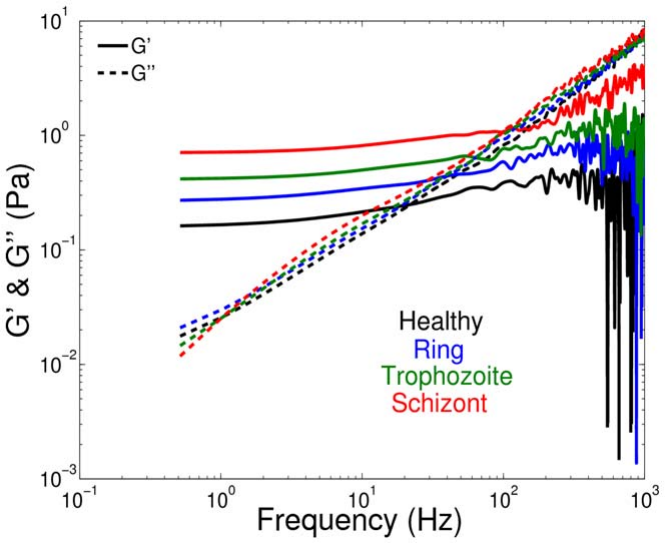
Components (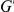 and 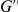) of the three-dimensional complex modulus for healthy RBCs and Pf-RBCs at different stages of parasite development. These measurements were obtained in simulations by monitoring membrane thermal fluctuations at 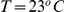. The shear moduli of Pf-RBCs for different stages are given in Table 1. The bending stiffness and the membrane viscosity in all cases are set to 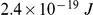 and 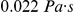, respectively.

In simulations an attached RBC is surrounded by a fluid of viscosity 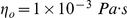 and is filled with a fluid of viscosity 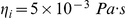, while the membrane viscosity is set to 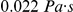. The mean square displacement 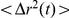 of several points on the RBC top is computed allowing us to derive 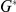 as follows [28]

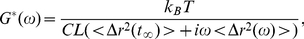
(13)where 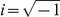, 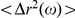 is the unilateral Fourier transform of 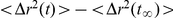, 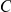 is a constant, and 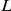 is a length scale. Here, we assume that 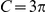 and 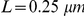 in agreement with those used in the experiments [23]. Figure 8 shows components of the complex modulus for healthy RBCs and Pf-RBCs at different malaria stages with the shear moduli from Table 1, 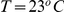. A similar increase in 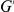, as that found for 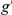 in the STC simulations above, is observed here since Pf-RBCs become stiffer during the consecutive stages of the parasite development. The loss modulus 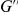 remains nearly the same for the progressing stages in agreement with the STC simulations. The complex modulus derived from membrane fluctuations of Pf-RBCs at different temperatures shows similar behavior as that from STC simulations. Agreement between the two-dimensional complex moduli from STC and the three-dimensional moduli from measurements of membrane fluctuations indicates an equivalence among these two rheological techniques.

### Pf-RBCs in Poiseuille flow

Experimental observations [29], [30] of a RBC flowing in tubes of diameter comparable with the RBC size showed a transition from biconcave to the parachute-like shape with increasing flow rate. Thus, at low pressure gradients the RBC retains its biconcave shape, while as the pressure gradient is increased the RBC transits into a parachute shape. Poiseuille flow in tubes can be characterized by the mean flow velocity defined as 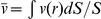, where 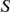 is the area of the tube cross-section, and 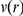 is the axial flow velocity.

**Figure 9:**
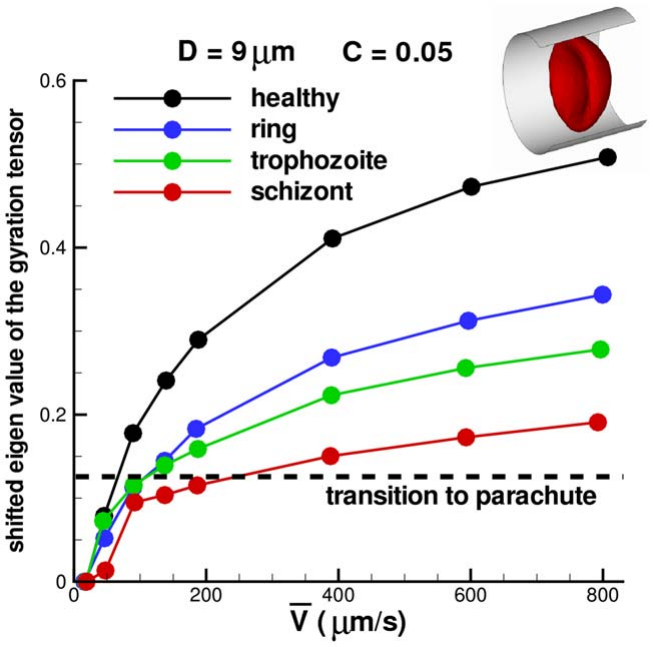
Shape representation of healthy RBCs and Pf-RBCs quantified by the shifted eigen-value of the gyration tensor at different stages of parasite development at 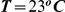 **.** The shift is done by subtracting the eigen-value of the equilibrium biconcave shape. 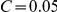 is the volume fraction of a single RBC. The shear moduli of Pf-RBCs for different stages are given in Table 1. The bending stiffness and the membrane viscosity in all cases are set to 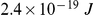 and 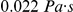, respectively.

A RBC in Poiseuille flow is simulated in a tube of the diameter 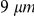. The biconcave-to-parachute transition can be identified by the smallest eigenvalue of the gyration tensor given as follows
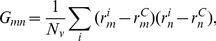
(14)where 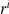 are the RBC vertex coordinates, 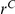 is the center-of-mass, and 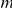, 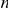 can be 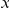, 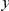, or 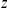. The eigenvalues of the gyration tensor characterize the RBC shape so that in equilibrium the smallest eigenvalue corresponds to the RBC thickness. At the biconcave-to-parachute transition the smallest eigenvalue becomes larger since the RBC elongates along the flow axes. Figure 9 shows the dependence of the shifted eigenvalue (the shift is done by subtracting the eigen-value of the equilibrium biconcave shape) of the gyration tensor for healthy and Pf-RBCs. The dashed line marks the biconcave-to-parachute transition as a function of the mean flow velocity. The transition for healthy RBCs occurs at a mean flow velocity of about 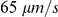. Pf-RBCs show the transition at higher flow rates with a nearly linear dependence of the transition on the shear modulus 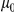. These results are in agreement with the numerical simulations of [11]. The shifted eigenvalues also show that stiffer cells are subject to a smaller cell elongation along the flow for the same mean velocity. Therefore, in comparison with softer RBCs, stiffer cells exhibit a higher flow resistance corresponding to an increase by several percent.

### Bulk viscosity of blood in malaria

The bulk viscosity of blood in malaria increases with the parasitemia level due to the increased stiffness of Pf-RBCs [21] with respect to healthy RBCs. This may lead to a significant increase of the stress on the body's cardiovascular system resulting in a reduced blood perfusion. Here, numerical simulations may offer a cheap and robust way to evaluate key properties of blood flow in microcirculation in malaria. The first estimates of the increase of blood flow resistance in microvessels in malaria were calculated in [14] predicting up to 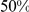 increase of flow resistance for high parasitemia levels.

**Figure 10:**
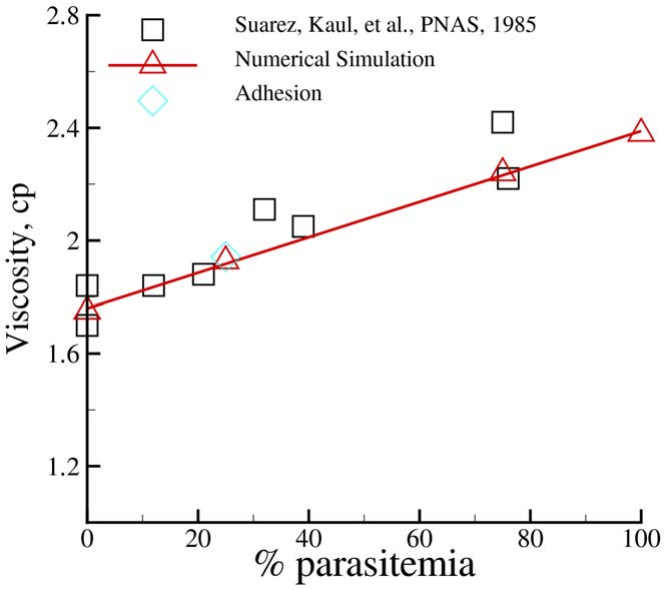
Viscosity of the infected blood in malaria for different parasitemia levels (percentage of infected RBCs with respect to the total number of cells in a unit volume) in comparison with experiments [**21**]. Infected blood is modeled as a suspension of healthy and Pf-RBCs at the schizont stage at 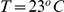 suspended in blood plasma. The shear moduli of Pf-RBCs for different stages are given in Table 1 and the bending stiffness is set to 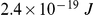. The diamond symbol represents a simulation with adhesive interactions among RBCs.

To further validate the Pf-RBC model we compute the bulk viscosity of infected blood for different parasitemia levels with hematocrit 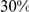 and shear rate 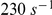 in accord with the experimental conditions in [21]. In the simulations, infected blood is modeled as a suspension of healthy and Pf-RBCs at the schizont stage at 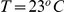 (see Table 1) in blood plasma. The homogeneous shear flow is modeled by the Lees-Edwards periodic boundary conditions [31] with the simulation domain size of 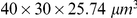 and with the total of 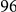 suspended RBCs. Figure 10 shows the bulk viscosity of infected blood for various parasitemia levels with respect to the experimental data [21]. The simulated viscosity as a function of the parasitemia is in excellent agreement with the corresponding experimental data, which show roughly linear dependence of the infected blood viscosity on parasitemia level. Note that the Pf-RBC model validated on a number of single cell experiments yields accurate prediction of the RBC suspension viscosity, which is a macroscopic characteristic of blood that depends on single-cell properties and their collective interactions.

Pf-RBCs at later stages of intra-erythrocytic parasite development are able to adhere to vascular endothelium and to other healthy and infected RBCs [32], [33]. Such adhesion between cells may contribute to the bulk blood viscosity in malaria. We performed a sensitivity study of the influence of adhesion interactions on the blood viscosity for the case of parasitemia 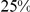. The adhesion interactions are mediated by the Morse potential (eq. (8)), which mimics RBC aggregation of healthy RBCs and adhesion between Pf-RBCs and other cells. The aggregation of healthy RBCs was achieved with the following parameters: 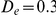, 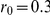, 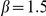, and 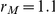 resulting in the maximum aggregation force between two cells to be in the range of 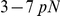. The adhesion interactions of Pf-RBCs were modeled with 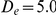, 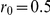, 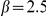, and 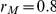 such that the maximum adhesion force between two cells is in the range of 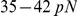. The simulation results at the shear rate of 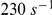 showed no significant increase in blood viscosity with respect to the adhesion interactions. This indicates that the simulated shear rate is high enough to disperse RBCs within the suspension and to diminish adhesion effects on the bulk viscosity. A stronger effect of adhesive interactions is expected at low shear rates. However, analogous simulations at low shear rates are much more expensive computationally, and thus they require substantial simulation efforts and will be the subject of future research.

## Discussion

The model we developed is able to properly capture the main biophysical characteristics and dynamic behavior of Pf-RBCs in malaria. The modeled mechanical properties are in excellent agreement with optical tweezers experiments [3] for various stages of intra-erythrocytic parasite development. Our simulations indicate that the RBC geometry is of importance in the cell stretching test and has to be closely modeled since Pf-RBCs at the schizont stage are often observed to have a near-spherical shape. The shear modulus of a near-spherical membrane is about 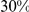 lower than that of the biconcave shape for the same uniaxial stress-strain response. A more deflated biconcave membrane may adjust its interior fluid volume in response to deformation, while a near-spherical shape is constrained by the fluid volume resulting in larger membrane strains. Analogously, a shape of a rigid parasite inside Pf-RBCs may affect membrane stretching and some results on adhesion dynamics are reported in [14], [34].

We also compared results of simulated twisting cytometry with optical magnetic twisting cytometry, which probes different stress states compared to optical tweezers. The membrane rheological characteristics obtained from the STC simulations showed discrepancies with respect to the OMTC experiments [5]. Several unresolved issues may contribute to the present discrepancies. First, the OMTC technique probes membrane properties *locally*, which may be non-isotropic resulting in deviations of the OMTC data. Second, a potential change in the membrane bending rigidity during the parasite development would greatly affect measurements of the storage modulus as shown for healthy RBCs in [13] and for Pf-RBCs in this paper. Third, the presence of the growing parasite inside Pf-RBCs may strongly influence the experimental measurements of the complex modulus especially for later intra-erythrocytic stages since the parasite volume becomes comparable with that of the RBC [4]. Finally, a change in the membrane or the internal fluid viscosity would affect measurements of the loss modulus.

STC rheological computations performed at different temperatures revealed even larger discrepancies with the OMTC experiments [5]. This points to possible fundamental differences between optical tweezers and OMTC that may induce even different heating. The experimental storage modulus of healthy RBCs shows a gradual increase with temperature elevation from 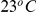 to 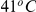 indicating continuous RBC stiffening. This is in contradiction with several other experiments (e.g., RBC micropipette aspiration [35], monitoring of membrane fluctuations [4]), where a gradual membrane softening with temperature increase was found. Other experiments (e.g., ektacytometry [36], optical tweezers [37]) have shown a slight increase in the shear modulus of healthy RBCs with temperature elevation, but statistical significance was not reached. Moreover, the STC simulations suggest that the increase of the shear modulus by 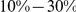 with changing temperature would result in a much smaller rise in 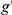 than that found in the experiments (see Figure 3 (left)). Hence, this discrepancy must be due to other changes in the membrane taking place at different temperatures. An increase in the membrane bending rigidity with increasing temperature would offer a possible explanation for the membrane's gradual stiffening found in the experiments. If we quantitatively follow the experimental data in Figure 3 (left), the RBC bending stiffness should increase from its value at room temperature to that at 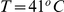 by a factor between three and four. However, several experiments on lipid vesicles [25], [26] showed a slight decrease in the membrane bending rigidity with increasing temperature suggesting the same to be likely true for RBCs. Marinkovic et al. [5] proposed a significant role of the entropic component to explain their experimental results since the RBC spectrin network is able to rearrange [38] under certain conditions such as metabolic activity or large strains, see also the new results in [27]. Another characteristic feature of the experimental data in Figure 3 is the increase of the loss modulus 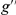 with increasing temperature indicating a rise in the membrane viscosity. The viscosity of liquids is known to decrease when temperature is elevated suggesting an analogous behavior for the liquid-like lipid membrane of RBCs. To understand the relevance of different contributions (e.g., RBC properties, metabolic activities) for the membrane rheology at different temperatures and Pf-RBC stages, more experimental and computational studies are required.

Membrane fluctuation measurements and predictions were shown to depend on cell geometry, on experimental or simulation conditions (e.g., adhesion strength, metabolic activity), and on membrane properties (e.g., shear modulus, bending rigidity). In addition, Figure 6 (left) showed that the fluctuations are not isotropic on the cell surface with smaller amplitudes in the RBC center and on the side. This can be partially explained by the RBC geometry since only height fluctuations were monitored. Moreover, a higher membrane curvature in central and side regions than that in the middle can contribute to damping of effective fluctuations. Finally, the presence of a rigid Pf parasite next to the Pf-RBC membrane may greatly affect fluctuation measurements. These findings suggest that local measurements of fluctuations would provide more accurate and detailed information and may yield a great opportunity to measure local Pf-RBC elastic and bending properties in experiments.

In Figure 7 we illustrated the complex behavior of membrane fluctuations for various temperatures and stages of the intra-cell parasite development. We have attempted to devise a correlation between membrane bending rigidity and the FWHM value for different temperatures, however no consistent dependency was found. Even though a large change in bending rigidity may potentially exist, it is more likely that other effects are involved. Membrane fluctuations may be influenced by metabolic activity such as the consumption of adenosine triphosphate (ATP) resulting in the spectrin network remodeling [38] and substantial enhancement of membrane undulations [39]. It is not clear whether metabolic activity was present in the experiments [4], nor whether it is actively triggered at the febrile temperature. In contrast, the experiments in [40] reported no dependence of membrane fluctuations on ATP. The most recent experiments of [27] shed light to this controversial point and identified the ATP activity to be a major factor contributing to about 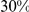 of membrane fluctuations, while the other 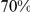 were attributed to thermal fluctuations. ATP metabolic activity results in spontaneous dissociations of the spectrin network at junctions with the lipid bilayer reducing tension within the cytoskeleton and yielding non-thermal membrane fluctuations. Park et al. [27] also showed that such active remodeling due to ATP is spatially inhomogeneous and may control local static and dynamic membrane characteristics, however it is still not clear whether the cytoskeleton rearrangement is of physiological importance. The observed complexity of membrane fluctuations requires more data from both experiments and simulations in order to quantify the simultaneous interplay of RBC membrane properties and metabolic activities at different temperatures and Pf-RBC stages. In addition, local measurements of fluctuations are preferred to eliminate the existing anisotropy discussed above and in [27].

Monitoring of membrane fluctuations also resulted in the complex modulus dependence similar to that obtained in the STC simulations. Here, rheological measurements are also greatly affected by potential changes in the membrane properties, by the presence of the Pf-parasite inside cells, and by the possible metabolic activities at different temperatures and Pf-RBC stages as discussed above.

The dynamics of Pf-RBCs in Poiseuille flow revealed a RBC transition to a parachute shape. The transition is governed by the membrane shear modulus, bending resistance (shown in [13]), and flow rate. The RBC dynamics may be additionally complicated by the presence of the rigid parasite, see [14], [34] with an example of adhesive dynamics. The computed flow resistance in case of a single Pf-RBC with respect to the analogous flow of pure plasma showed a slight increase by only several percent, while the membrane shear modulus was increased up to ten times. Such a small difference in the flow resistance is attributed to a very low cell concentration with the effective volume fraction of 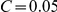. For higher RBC volume fractions blood viscosity in malaria is more sensitive to a change in the RBC membrane properties, which was shown in the simulations that accurately predict bulk viscosity of infected blood for parasitemia levels up to 100%. In addition, simulations with adhesive interactions between RBCs showed that this contribution to bulk viscosity is negligible for the relatively high shear rates. At low shear rates the effect of adhesive interactions is expected to be more pronounced, however such simulations pose significant computational challenges. Simulations at low shear rates are much more expensive because they require much longer time for a system to equilibrate and to reliably measure very small shear stresses.

Finally, it is worthwhile to discuss limitations of the current model with respect to its application in malaria. Despite of the successful modeling of Pf-RBC membrane deformation in the stretching tests, the results for cell rheology show considerable discrepancies in comparison with the experimental data. Recent experiments on RBC fluctuations [27] provide evidence that membrane flickering may be complicated by non-isotropic cell properties and by active processes such as spectrin network remodeling due to the metabolic activity. Clearly, the purely mechanistic membrane model with isotropic properties is able to capture only non-active thermal fluctuations, while full models that take into account active cell properties have to be developed. An additional property of Pf-RBCs, which was not taken into account in the current model, is the presence of a parasite inside the cell. The effect of the parasite is expected to be small at the earlier stages (e.g. ring stage) of the intra-cell development, however the parasite effect is likely to become non-negligible at the later stages (trophozoite and schizont). The first attempts of explicit modeling of the parasite show such effects for the cases of Pf-RBC adhesive dynamics [14], [34] and microflow cytometer [41]. Despite of these encouraging results, modeling with an explicit parasite representation is limited due to the lack of available experimental data on the parasite size, geometry, and interactions with a membrane for various stages. Such experimental studies will be of great interest for further development of truly predictive models. Nevertheless, the current model appears to be very promising for blood flow simulations in malaria, since it was able to accurately capture the bulk blood viscosity. This indicates that the aforementioned processes and features not taken into account in the present model may be negligible in hydrodynamic flow simulations such as blood flow in malaria. In conclusion, one has to be aware of the limitations and advantages that a particular model possesses to apply it to the problems of interest in malaria. However, such DPD modeling combined with new deformability-based microflow cytometers [41] that can characterize the properties of individual Pf-RBCs *in vitro* could provide a systematic approach for predicting the collective biorheological behavior of infected blood in malaria.
